# Supplementary material for: Integrated Somatic and Germline Whole-Exome Sequencing Analysis in Women with Lung Cancer after a Previous Breast Cancer
Source: Cancers (Basel). 2019 Mar 28;11(4):441. doi: 10.3390/cancers11040441 (PMC6520745; doi:10.3390/cancers11040441)
Supplement: Supplementary file 1 [file cancers-11-00441-s001.zip › cancers-452240-Supplementary/Figure S1.pdf]

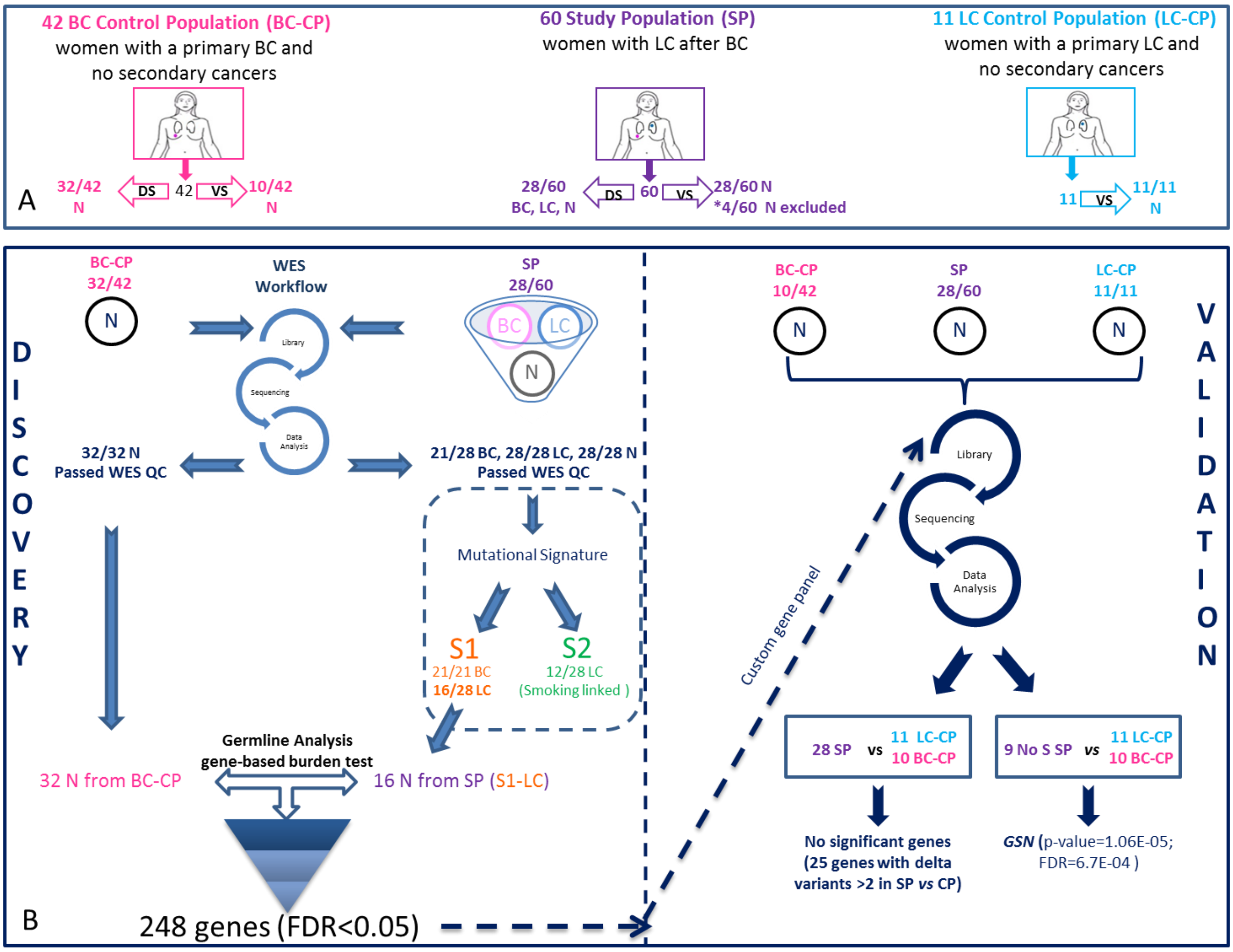

Figure S1: Flow chart of study design. The flow chart reports the number of patients (Study Population: SP and Control Population: CP) and types of samples (Breast Cancer: BC; Lung Cancer: LC; Normal tissue: N) (Panel **A**) involved in each analysis (whole exome sequencing: WES; next generation sequencing (NGS) custom panel) (Panel **B**).

Abbreviations: DS: discovery set; FDR: false discovery rate; QC: quality control; S: mutational signature; vs: versus; VS: validation set; WES: whole exome sequencing.
