# Supplementary material for: Integrated Somatic and Germline Whole-Exome Sequencing Analysis in Women with Lung Cancer after a Previous Breast Cancer
Source: Cancers (Basel). 2019 Mar 28;11(4):441. doi: 10.3390/cancers11040441 (PMC6520745; doi:10.3390/cancers11040441)
Supplement: Supplementary file 1 [file cancers-11-00441-s001.zip › cancers-452240-Supplementary/Figure S2.pdf]

2A

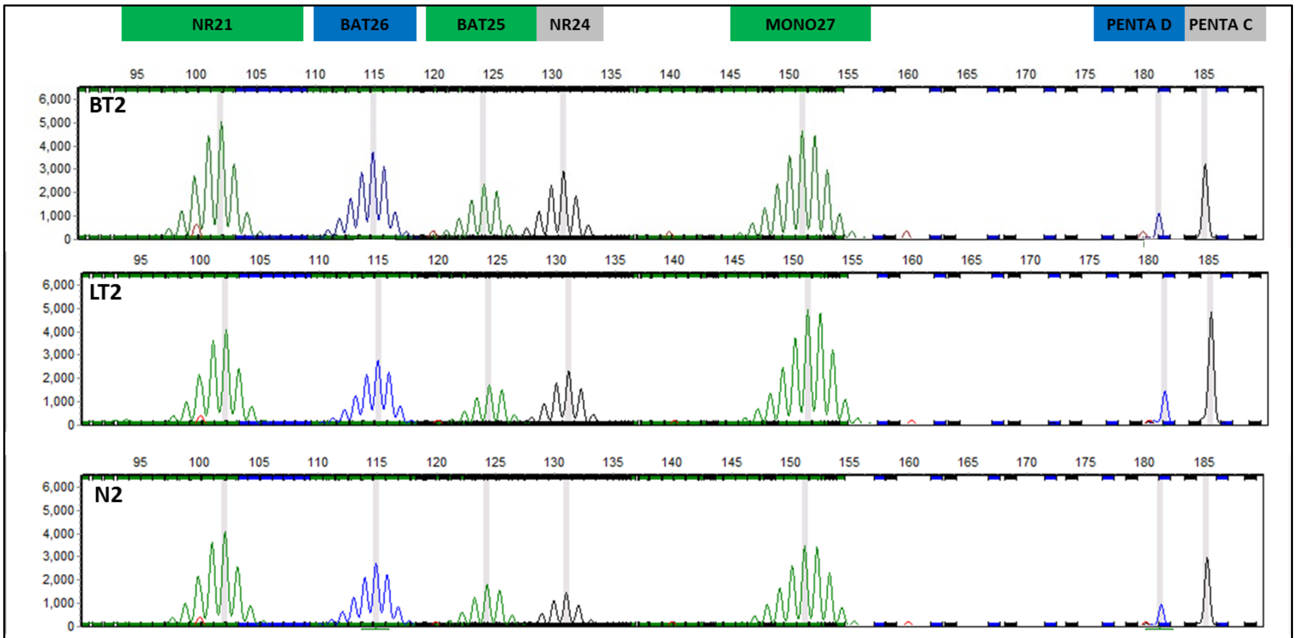

Sample	NR21		BAT26		BAT25		NR24		MONO27		PENTA D		PENTA C	
	Size 1	Height 1	Size 1	Height 1	Size 1	Height 1	Size 1	Height 1	Size 1	Height 1	Size 1	Height 1	Size 1	Height 1
BT2	102.1	5032	114.9	3707	124.2	2379	130.9	2901	151.1	4632	181.2	1107	185.1	3243
LT2	102.1	4096	115.0	2765	124.3	1715	131.0	2334	151.2	4914	181.3	1453	185.2	4818
N2	102.1	4087	114.9	2746	124.3	1792	131.0	1444	151.2	3455	181.3	953	185.2	2961

2B

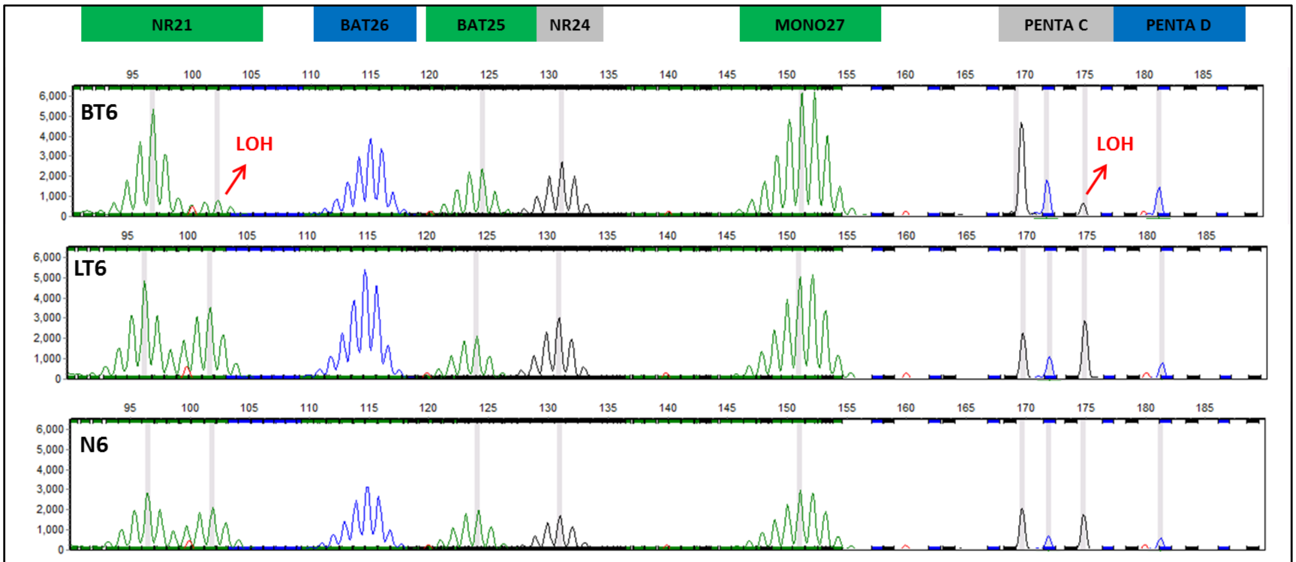

Sample	NR21				BAT26		BAT25		NR24		MONO27		PENTA C				PENTA D			
	Size 1	Size 2	Height 1	Height 2	Size 1	Height 1	Size 1	Height 1	Size 1	Height 1	Size 1	Height 1	Size 1	Size 2	Height 1	Height 2	Size 1	Size 2	Height 1	Height 2
BT6	96.7	102.1	5362	777	115.0	3882	124.4	2386	131.1	2713	151.3	6200	169.7	174.9	4696	661	171.8	181.3	1794	1454
LT6	96.4	101.9	4833	3551	114.8	5388	124.1	2110	131.0	3015	151.0	5125	169.7	174.8	2253	2853	171.9	181.3	1086	785
N6	96.5	101.9	2842	2127	114.8	3122	124.1	1968	131.0	1736	151.1	2812	169.7	174.8	2042	1750	171.9	181.3	681	609

Figure S2. Fragment analysis in microsatellite instability. **S2A** Example of a normal asset (no MSI) for all markers in both tumors (BC and LC) compared to the normal tissue. **S2B** Example of allele imbalances in a BC sample.
